# Polarization‐Controlled Transmissive Plasmonic Color Filter Using a Dimer‐Aperture Array

**DOI:** 10.1002/advs.202501941

**Published:** 2025-03-24

**Authors:** Shuhao Wu, Peter W.R. Connolly, Vincenzo Pusino, Gerald S. Buller, David R.S. Cumming

**Affiliations:** ^1^ James Watt School of Engineering University of Glasgow Glasgow G12 8QQ UK; ^2^ School of Engineering and Physical Sciences Heriot‐Watt University Edinburgh EH14 4AS UK

**Keywords:** metasurface spectral filter, plasmonic, polarization control, structural color

## Abstract

Complex color and polarization selective technologies are of increasing importance in scientific, security, and commercial imaging applications. A new dimeric plasmonic filter structure based on periodic aperture arrays is reported to provide an effective method for making planar color‐selective structures by exploiting the properties of extraordinary optical transmission in thin metal films. The visible band transmission‐mode polarization‐dependent color filters reported in this work exploit only a single layer of aluminum patterned using a hexagonally periodic dimer‐ellipse aperture structure. It is shown experimentally that the structure exhibits a minimum extinction ratio of over 20, 100, and 150 for red, green, and blue channels respectively, and a peak transmission of over 30%. It is demonstrated that dual images can be encoded using polarization selectivity into a single structure. The fidelity of the method is demonstrated with micro‐scale reproductions of complex artworks showing the ability to reproduce 76% of the sRGB color gamut with polarization selectivity. The structure can be readily fabricated with only a single‐step lithography and etching process, so that the technique may be widely used.

## Graphical abstract

An aluminum dimer aperture array plasmonic metasurface is proposed and fabricated to achieve efficient polarization‐color filtering with CMOS‐compatible materials. The structure's simultaneous polarization‐color filtering capability is shown using structural color generation, where color gamut coverage of 76% and extinction ratio over 20 is achieved. Image encryption and micro‐scale reproduction of color‐rich paintings are demonstrated using the structure.

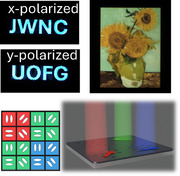

## 1 Introduction

**Figure 1:**
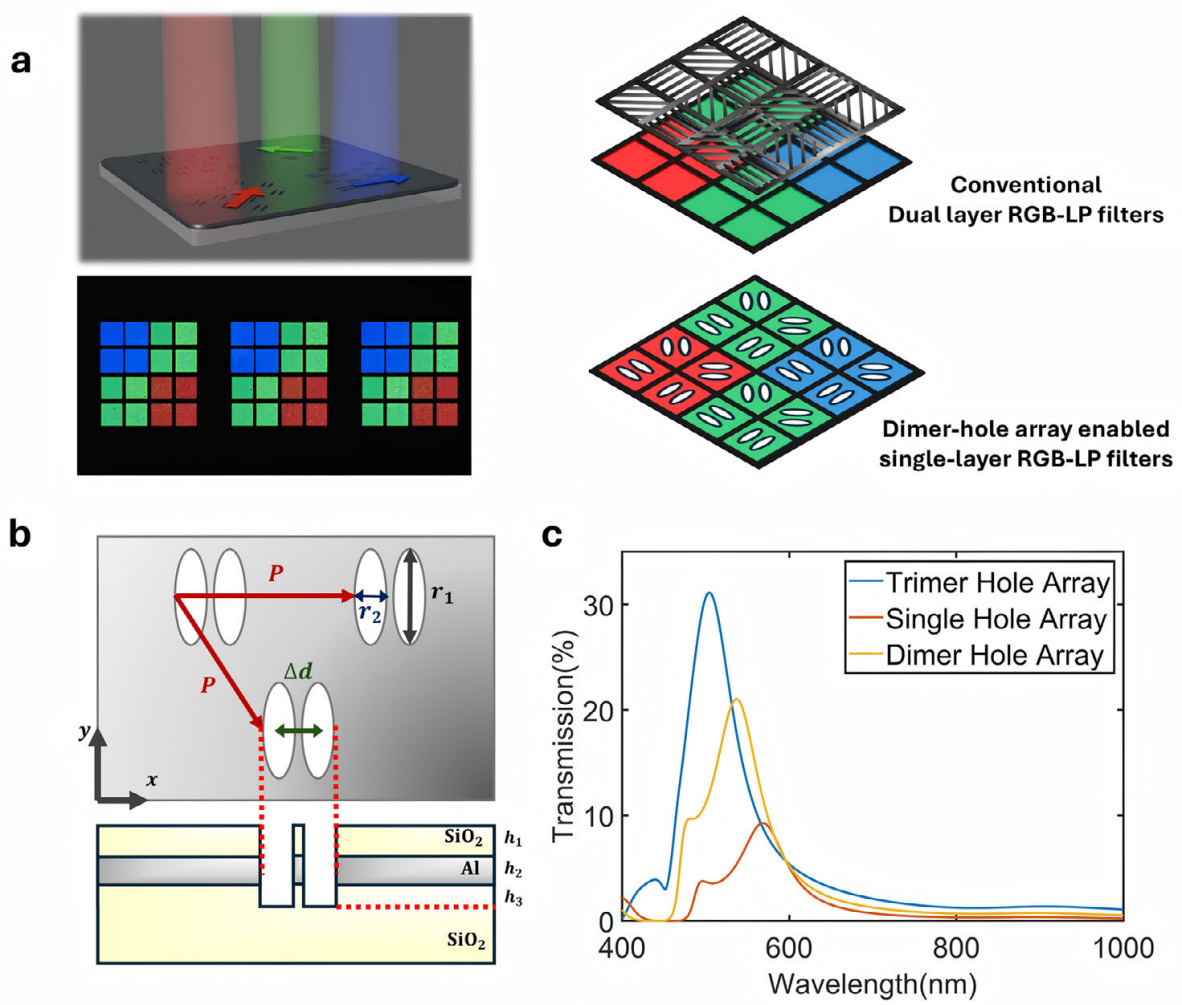
Design of the proposed polarization‐controlled transmissive aluminum plasmonic metasurface filter. a) An illustration of the filter function and optical photomicrograph of fabricated RGB‐LP array (under unpolarized illumination). The size of the color filter pixel shown here is 10 µm × 10 µm. The metasurface serves as both a linear polarizer and a color filter with a single‐layer periodic aperture in an aluminum film. b) The geometrical structure of the metasurface filter. *P* refers to the period of the triangular lattice, while *r*_1_, *r*_2_ refers to the major and minor axes of the ellipse. Δ*d* = *P* × *g* refers to the distance between the dimer apertures. The dimensions are *h*_1_ = 150 nm, *h*_2_ = 150 nm, *h*_3_ =  100 nm. c) Transmission enhancement of the multi‐elliptical aperture array. Multiple aperture arrays enhanced the transmission without broadening the linewidth of color filtering.

The color or spectral response of an object at visible wavelengths (400–700 nm) provides important image information for humans and computer vision alike. Polarization, another important property of light, is less perceptible but contains crucial information such as the reflectivity of surface contours. Nature has discovered methods of imaging with polarization,^[^ ^1^, ^2^ ^]^ and working in tandem with color selectivity to deliver an information‐rich vista. Polarization sensitivity is important to many vision tasks including passive underwater imaging,^[^ ^3^ ^]^ target contour enhancement for 3D imaging,^[^ ^4^ ^]^ image dehazing,^[^ ^5^ ^]^ and biomedical imaging.^[^ ^6^ ^]^ Imaging systems that simultaneously resolve polarization and color have consequently attracted the interest of many researchers and industry.^[^ ^7^, ^8^, ^9^, ^10^ ^]^ Typically, existing color‐polarization cameras use a dual‐layer structure for filtering, as shown in **Figure** **1**a, employing a thin film system for color filtering, and an additional grating layer for polarization filtering, that requires complex fabrication and integration. To improve system integration and performance, it is of considerable interest to exploit the properties of the emerging capabilities of metasurfaces. In addition to enabling multiple complex optical functions to be readily deployed on a single substrate, metasurfaces have the additional advantage that they can be fabricated with complementary metal oxide semiconductor (CMOS)‐compatible processes and may be further integrated on to the surface of image sensors to make compact polarization‐color filtering systems.

Metasurface‐based color filters have been extensively reported.^[^ ^11^, ^12^, ^13^, ^14^, ^15^, ^16^, ^17^, ^18^, ^19^, ^20^ ^]^ They modulate the optical wavefront using quasi‐planer optical structures to achieve efficient color filtering. High‐brightness and large‐saturation spectral filters based on all‐dielectric resonance metasurface have drawn widespread attention.^[^ ^14^, ^16^, ^18^, ^19^, ^21^, ^22^, ^23^ ^]^ All‐dielectric structures have a natural efficiency advantage over metallic/plasmonic structures in structural color generation owing to low loss, enabling high‐efficiency and narrow linewidth color filtering. Some of these structures rely on collective resonance modes, such as multi‐level Mie resonance, guided‐mode resonance, and quasi‐bound states in the continuum (q‐BIC) modes. These techniques require a large number of periods to achieve mode generation and enhancement.^[^ ^17^ ^]^ It has been reported that a TiO_2_ metasurface can reduce the periodicity dependence of a structure by using magnetic dipole (MD) modes generated by a single resonator to achieve a high spatial resolution color filter.^[^ ^19^ ^]^ Using a semiconductor material such as silicon as a resonator can also help generate angle‐insensitive MD under s‐polarization. By further introducing a refractive index matching layer, such resonators have been made to show high color saturation and excellent spatial resolution color filtering.^[^ ^18^ ^]^ Despite the vivid colors that have been demonstrated with structures working in reflection mode, apart from studies of the subtractive‐color mode,^[^ ^24^, ^25^, ^26^ ^]^ there are limited experimental reports on transmission mode metasurface color filters. Without increasing computational costs, transmission‐mode metasurface filters offer a direct and simple integration advantage for color imaging systems. Some of these filters also feature CMOS‐compatible materials and fabrication processes,^[^ ^11^, ^27^, ^28^, ^29^ ^]^ making them a potential replacement for dye‐based or thin‐film‐based Bayer filter structures that are dominant in CMOS‐image sensor (CIS) filter systems.

Color filters based on plasmonic metasurfaces with metallic apertures can readily operate within several periods hence they are a good match to the typical pixel size (micron level) of an imaging array detector. They are therefore a good alternative for filter structures of color cameras.^[^ ^15^, ^26^, ^30^, ^31^ ^]^ Such plasmonic filter arrays have also been demonstrated for use in fluorescence imaging^[^ ^32^ ^]^ and video‐rate single‐photon imaging.^[^ ^33^ ^]^ Polarization‐sensitive color filtering can be achieved through the anisotropic design of aperture shapes or array lattice vectors,^[^ ^34^, ^35^, ^36^ ^]^ especially through the anisotropy of aperture dimensions. Plasmonic filters based on noble metals like gold and silver have been widely reported in the visible and near‐infrared regions due to their low Ohmic losses.^[^ ^30^, ^37^, ^38^ ^]^ However, the interband transition of gold hinders its color rendering below 500 nm (corresponding to the blue spectral range). Silver nanostructures, on the other hand, are prone to sulfidation. Neither gold nor silver are compatible with CMOS technology,^[^ ^27^ ^]^ limiting their practical application. Consequently, aluminum‐based plasmonic filters that offer low material cost and ease of fabrication, have long attracted significant attention.^[^ ^12^, ^36^, ^39^, ^40^ ^]^ However, the low Q‐factor resonance of aluminum plasmonic filters is undesirable for direct color reconstruction. Another problem is that relying on the anisotropic design of aperture structures or array lattice vectors to achieve polarization‐sensitive response requires a trade‐off between peak transmission, linewidth, and polarization extinction ratio. Current structures can only achieve different polarization responses,^[^ ^41^, ^42^, ^43^ ^]^ but it remains a challenging issue to realize a polarizer with a high extinction ratio. It is therefore necessary to investigate how to obtain a narrow linewidth and high polarization extinction ratio without sacrificing peak transmission for aluminum spectral‐polarization filters.

In this work, we propose and experimentally demonstrate a plasmonic metasurface with a dimer‐elliptical aluminum aperture array structure, as shown in Figure 1a. Two specific designs have been made to improve aluminum plasmonic filter spectral linewidth and polarization extinction ratio without excessively sacrificing peak transmission. The first design innovation is the introduction of a dimer structure that helps double the peak transmission while maintaining the polarization extinction ratio when compared to a monomer‐aperture configuration. The second design introduction is a deep etching of the SiO_2_ substrate to reduce the linewidth while only slightly sacrificing the peak transmission (difference < 5%). The introduction of these two designs allows the proposed polarization‐sensitive color filter to achieve a peak experimental peak transmission of ≈30%, a polarization extinction ratio comparable to commercialized polarization filters for the blue channel (>150 @ 465 nm), and over 76% RGB color gamut coverage (CIE 1931, 2° observer).

To demonstrate the precise polarization control and structural color generation capabilities of the proposed structure, we have experimentally shown micrometer‐scale color image generation and polarization‐encoded images using the proposed transmissive filter in a coded‐array format. The fabrication of the structure requires only a single lithography exposure and chlorine‐based dry etch.^[^ ^44^ ^]^ While this paper uses electron‐beam lithography (EBL) for concept demonstration, the dimensions used are such that it could be replaced with photolithography or nanoimprinting for large‐scale manufacturing.

## 2 Design and Modelling

### 2.1 Color Filtering

The phenomenon of extraordinary optical transmission (EOT) has been successfully used to describe and hence fabricate transmissive filters.^[^ ^27^, ^31^, ^37^, ^39^, ^40^, ^45^, ^46^ ^]^ Such filters consist of an array of small apertures etched into a thin metal film. When the periodic lattice vector of the apertures compensates the wave vector of the surface plasmon polariton (SPP) at normal incidence, the wavelength, denoted as λ_SPP_, can serve as an indicator for the transmission window position,^[^ ^31^ ^]^ given by Equation (1):

(1) $$\lambda_{SPP}=\frac{p}{\sqrt{\frac{4}{3}\left(i^{2}+ij+j^{2}\right)}}\sqrt{\frac{\varepsilon_{m}\varepsilon_{d}}{\varepsilon_{m}+\varepsilon_{d}}}$$

where (*i*, *j*) is the diffraction order and ε_*m*_ and ε_*d*_ are the permittivity of the metal and the dielectric immediately adjacent to the metal, respectively. A triangular lattice was chosen because it provides a denser arrangement compared to a square lattice and a larger separation between (1, 0) and (1, 1) order λ_SPP_, which helps achieve higher‐quality color filtering that shows better out‐of‐band rejection. In this work, we designed a filter using the (1, 0) mode.

In previous structures and models, metal periodic aperture arrays were typically constructed directly onto a substrate to make a diffraction grating structure. A grating made in this way provides an additional wave vector that aids the incident wave propagating from the substrate to couple into the SPP. It then interferes destructively or constructively with the directly transmitted mode radiating into free space. Constructive interference between the SPP wave and the directly transmitted mode corresponds to a maximum in the far‐field transmission spectrum. Although the actual transmission maximum will exhibit a red shift due to the additional phase shift in the apertures,^[^ ^47^, ^48^ ^]^ Equation (1) gives a good indication of the position of the rising edge of the transmission peak. Increasing the aperture radius increases the transmission at all wavelengths, hence increasing the peak transmission. However, with an SPP mode, the field intensity is greatest at the metal edge of the apertures. Since the aperture area increases quadratically with radius, substantially more light not associated with the SPP mode is transmitted in larger apertures resulting in a broadened linewidth.

### 2.2 Polarization Filtering

**Figure 2:**
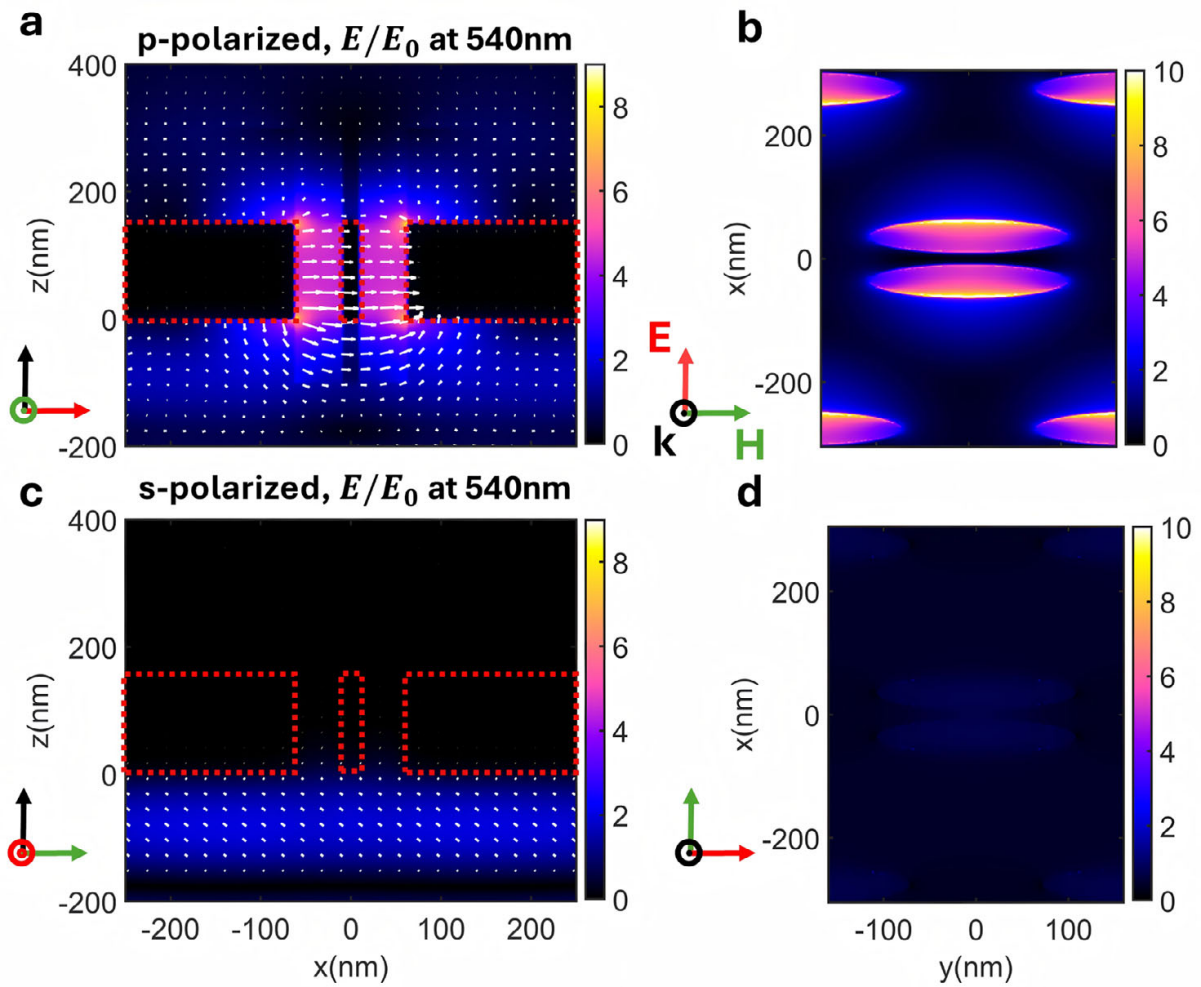
x‐z view (side view) and y‐x view (top view of the dielectric‐metal interface) of the normalized electric field distribution for a green filter for normal incidence. The orientation of the electric field, magnetic field, and the wavevector of the incident light are noted in red, green, and black respectively. a,b) With TM‐polarized light incidence (w.r.t. x‐z plane). A strong field enhancement exists at the edge of the ellipse largely as a consequence of SPP modes. c,d) With TE‐polarized light incidence. Most light is blocked by the metal film and no field enhancement is observed.

It has been previously shown that the excitation of SPPs is closely related to the polarization of the external incident field,^[^ ^31^, ^34^, ^35^, ^47^, ^48^ ^]^ and the formation of SPP in EOT is primarily concentrated at the edges of the apertures. As a result, asymmetric shapes of the metal apertures (corresponding to asymmetric edge lengths in the x‐y direction) or arrays with symmetry‐breaking periods will cause the transmission spectrum to be sensitive to the polarization direction of the incident wave. As shown in **Figure** **2**a,b, there is field enhancement at the metal aperture edges, and strong excitation occurs only when the magnetic field is aligned along the major axis of the elliptical apertures (perpendicular to the minor axis).

Thus, by adjusting the orientation of the major axis of elliptical apertures, the polarization direction of the filter can be correspondingly tuned. Increasing the metal thickness enhances the mechanisms above but with increased Ohmic losses. Taking this trade‐off into account, we have set the metal thickness to 150 nm where the peak transmission remains above 20%.

## 3 Results and Discussion

### 3.1 Finite Size and Incident Angle Tolerance Analysis

**Figure 3:**
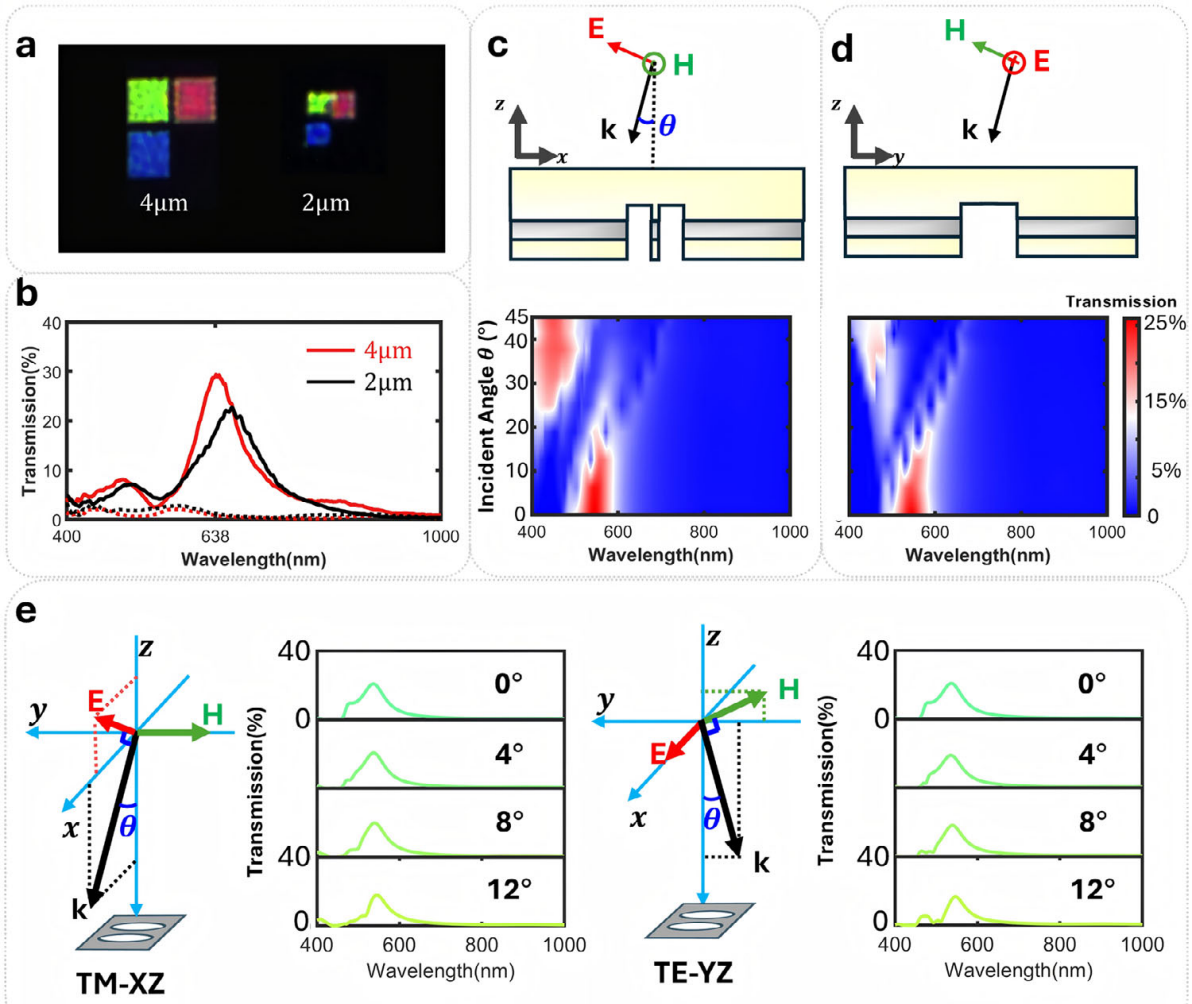
Finite‐sized array and angled incidence tolerance check. a,b) Photomicrographs of fabricated 4 µm and 2 µm sized filter pixels and the measured spectrum for red filters. Dashed lines show TM‐polarized incidence (w.r.t. x‐z plane) while solid lines show TE‐polarized incidence. c) The effect of changing the angle of incidence θ for TM‐polarized incidence with respect to x‐z plane and in the wavelength range 400–1000 nm. d) The effect of changing θ for TE‐polarized incidence with respect to y‐z plane. e) The detailed transmission spectrum for the angle of incidence within 12 degrees.

To demonstrate the feasibility of using the proposed structure as a focal plane array polarization‐color filter layer for imaging applications, its color‐polarization filtering performance at different pixel sizes is especially important. Since our device is based on the SPP process, which is a non‐local mode, it is expected that the filtering performance will degrade as the number of periods decreases. To investigate the degradation, we fabricated and tested filters with small finite sizes, ≈2 and 4 µm sized square pixels, which correspond to roughly three and six periods for red filters (we have rounded the number of periods for given size to lower integral in fabrication), respectively. We chose the red filter as a reference because the red wavelength filters (≈640 nm) correspond to the largest period, meaning that they should be affected by the finite size limitation before the blue and green filters for small pixels. As shown in **Figure** **3**a we found that for the 2 µm size, there was some performance degradation observed in the spectral response, but that the structure still exhibited distinct red wavelength filtering characteristics. This is sufficient for most industrial CMOS camera pixel sizes.^[^ ^49^ ^]^ Moreover, it can also be combined with high‐performance detectors, such as single photon avalanche diode detectors (SPADs) that have so far been demonstrated with a minimum detector pixel size of ≈8 µm to achieve simultaneous polarization and color reconstruction under low‐light‐level conditions.^[^ ^10^ ^]^

For many imaging applications, the incidence angle response of the color filter is particularly important. We have therefore examined the response of the proposed detector under different incident angles, as shown in Figure 3b. We focused on the case where the magnetic component and the Poynting vector lie in the plane parallel to the long axis of the ellipse, which corresponds to the condition with the maximum polarization transmission angle.

From the oblique angle response of the green filter in Figure 3c–e, it can be observed that whether the light incidence is TM polarized (w.r.t. xz plane) or TE polarized (w.r.t. yz plane), the filter maintains good color filtering performance within an incident angle range of 0–10°. However, when the incident angle exceeds 15°, the peak wavelength experiences a redshift, and higher‐order peaks appear in the short wavelength region. For incident angles up to 15° the polarization extinction ratio of the transmitted light is never less than 10^5^ in simulation. As a consequence of these off‐axis properties the proposed filters can be used for imaging applications without large color distortion with F‐number down to f/2.

### 3.2 Color—Polarization Palette

**Figure 4:**
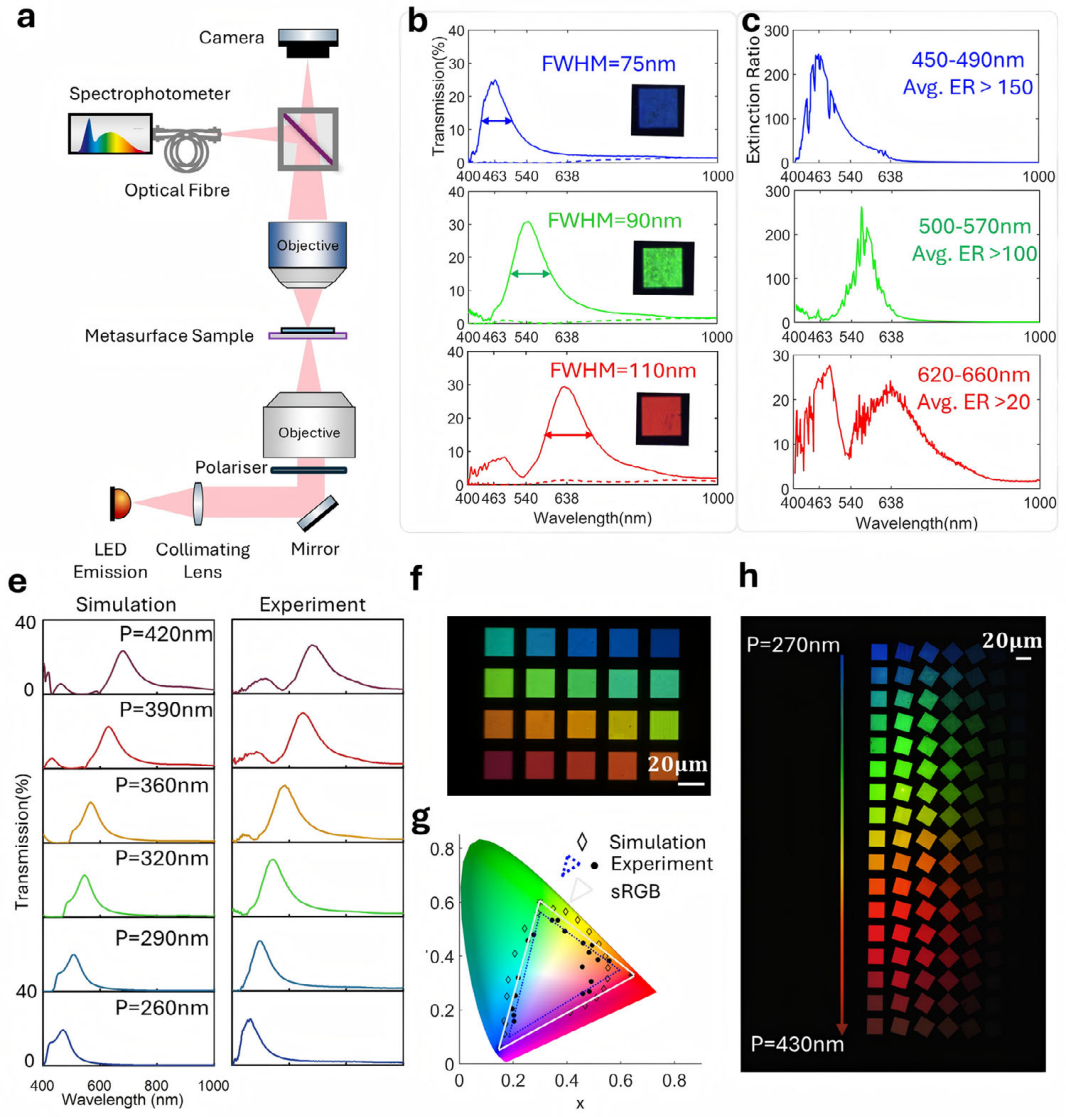
Spectral characterization results of the fabricated dimer plasmonic metasurface sample. a) Optical path for sample spectral characterization. The sample was tested in a micro‐spectrophotometer in the Köhler illumination configuration, where a polarizer is settled underneath the condenser. The condenser has a numerical aperture of 0.5 and all spectra were obtained using an objective with 20× magnification. b) Transmission spectrum under X‐polarized (E‐component perpendicular to major axis, solid lines) and Y‐polarized linear polarization (E‐component parallel to major axis, dashed lines) of RGB filters. c) Calculated extinction ratio within the full spectrum. As the spectrometer readings got unstable for very low‐intensity illumination, we rounded all transmissions smaller than 0.1–0.1% when calculating the extinction ratio. e) Simulated and tested transmission spectrum for filters with periods ranging from 240 to 440 nm, 6 out of 20 are shown here. f,g) Color palette under the microscope of the 20 filters and their calculated position on CIE 1931 axis. h) Color‐polarization palette of 20 filters under X‐polarization illumination.

Based on the color‐polarization filtering performance principles described in Section 2, we fabricated 20 µm  × 20 µm RGB filters as proof‐of‐concept. The filtering performance and polarization extinction ratios of the resulting components are shown in **Figure** **4**b,c. The spectra were obtained from the test optical path shown in Figure 4a, where a microscope with a fiber‐coupled spectrometer was used to characterize a micro‐scale metasurface area. The experimentally measured transmission spectra of the RGB filters under normal incidence demonstrated peak transmission of ≈25% (B), 30% (G), and 30% (R) at wavelengths of 465, 540, and 638 nm, respectively, with a full width at half maximum (FWHM) of ≈75, 90, and 110 nm for the blue, green, and red filters. In addition, when the illumination is polarized rotating from 0° to 90° with respect to the minor axis of the ellipse, the filters exhibit an extinction ratio of over 150, 100, and 20 for the blue, green, and red channels, respectively. The full spectrum extinction ratios are shown in Figure 4c.

In the green and red channels, the higher‐than‐simulated transmission coefficient and lower‐than‐simulated extinction ratio can be attributed to fabrication errors and test methods, as discussed in detail in Section S5 (Supporting Information).

To demonstrate the geometric tunability of the proposed structure in color filtering, we fabricated 20 filters by scaling the periodicity while keeping other proportional parameters unchanged. The periods of these 20 filters ranged from 240 to 440 nm, with a step size of 10 nm. Their corresponding simulation and experimental results are shown in Figure 4e. Under illumination at the polarization angle corresponding to the maximum transmission, the transmission spectra of these filters cover an area that occupies ≈76% of the sRGB color space in the CIE 1931 color diagram. We also fabricated a color palette with a finer step size (8 nm, ranging from 270 to 430 nm) to visually display the polarization‐color filtering performance, as is shown in Figure 4h. A broader white light polarization filter can be achieved by increasing the relative size of the ellipse's long axis.

### 3.3 Image Encryption

**Figure 5:**
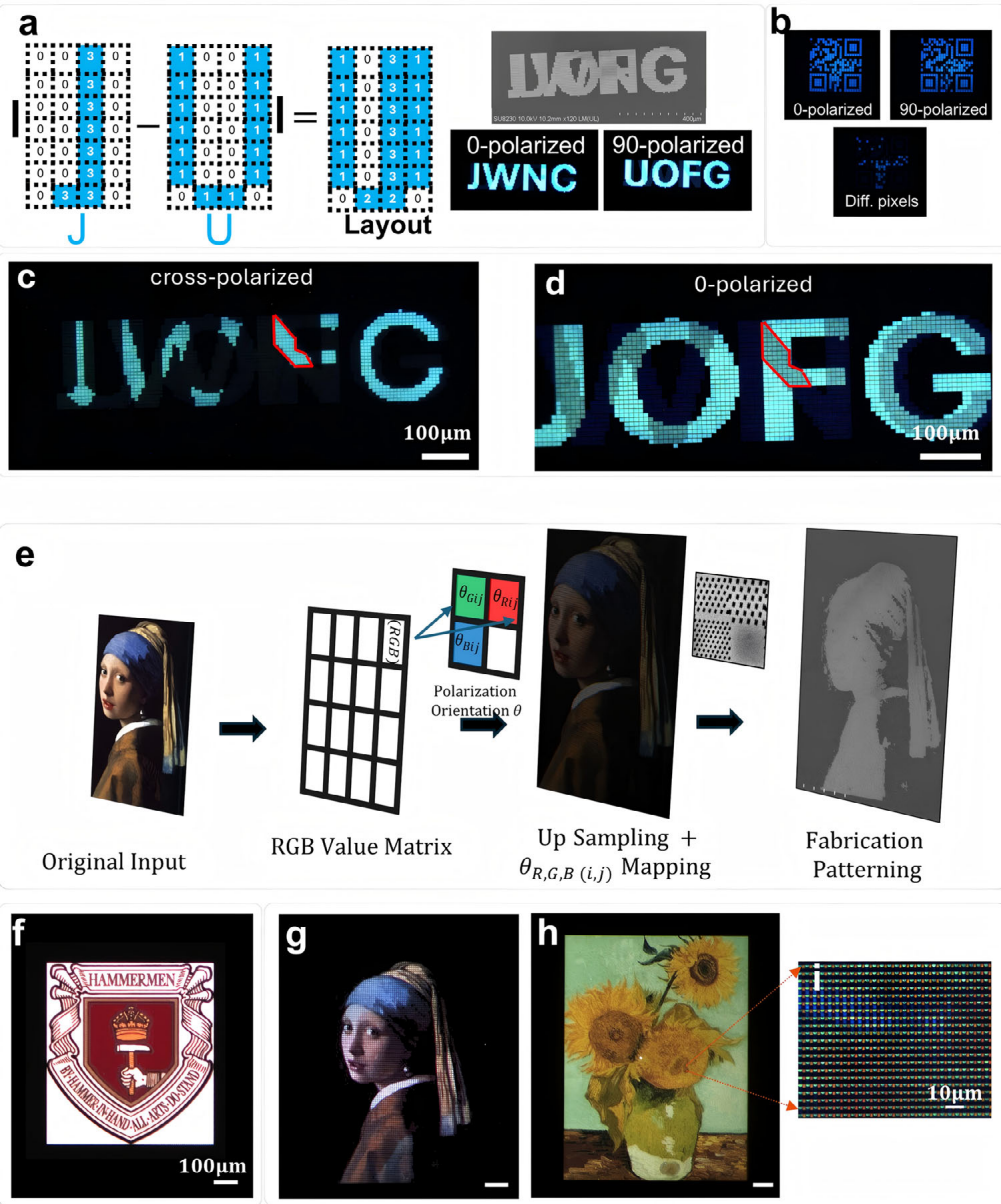
Demonstration of color‐polarization filtering function of the proposed metasurface. a) Orthogonal linear‐polarization encrypted dual‐state display. The pixels were encoded with (0,1,2,3) in the final layout, representing no transmission, 0‐degree polarization angle, 45‐degree polarization angle, and 90‐degree polarization angle respectively. b) Polarization‐color encrypted bar‐coding. Two different bar codes were encrypted in one layout, while only under blue illumination with a 0‐degree polarization angle will the right bar code appear. c,d) A micrograph at higher magnification of the letters when viewed using cross‐polarized imaging (polarizer and analyzer aligned along 0‐ and 90‐degree respectively) and 0‐polarized imaging. The cross‐polarized image shows the 45‐degree polarization filters, which resemble the area showing weaker intensity in (d). e) Working principle for producing micro‐scale paintings based on color‐mixing with proposed color‐polarization filter. The RGB value of the original input was read, and each pixel was up sampled to a 2 × 2 block, respectively representing R, G, B channel and a black channel (no transmission). Then the RGB values were normalized and mapped by polarization angles, which were then fabricated through E‐beam lithography and dry‐etch. f–h) Photomicrograph of the fabricated micro‐paintings under polarized illumination. The scale bars are 100 µm for all paintings (The Crest of the Hammermen of Glasgow in f, Girl with a Pearl Earring in g, Sunflower in (h). i) Zoom‐in view of the upper‐right part of painting h, each color pixel was in 2 × 2 µm format.

To visually demonstrate the polarization filtering capabilities of the proposed metasurface, we applied the encoding process shown in the figure to orthogonally polarized letters “JWNC” (James Watt Nanofabrication Centre) and “UOFG” (University of Glasgow). In the final layout, the numbers 0, 1, 2, and 3 represent pixels with no pattern (transmission = 0), pixels oriented at 0° linear polarization, 45° linear polarization, and 90° linear polarization, respectively. Each pixel was 8 µm × 8 µm. Light transmitted through the 45° linear polarization filter will be seen at the same intensity for 0 and 90° incidence. After fabrication, the layout reveals different patterns when illuminated with orthogonally polarized light. **Figure** **5**c shows the cross‐polarized microscopic image of the letters. Using a polarizer set at 0° orientation for illumination and an analyzer set at 90° orientation, only 45° filters could be imaged.

The same encoding and fabrication process can be used for 2D orthogonal polarization encoding, which could be applied to anticounterfeiting displays operating in transmission mode using QR codes. We experimentally fabricated a polarization encrypted bar‐code shown in Figure 5b to demonstrate this concept. As before, the size of each functional filter pixel was 8 µm  ×  8 µm. The photograph of different pixels (noted as Diff. pixels) was taken using cross‐polarized microscopy, where a polarizer was set at 45° orientation, and an analyzer was set at 135° orientation.

### 3.4 Simultaneous Color‐Polarization Filtering Enabled Micro‐Scale Paintings

Finally, we demonstrate the simultaneous polarization and color filtering performance of our filters through micro‐scale paintings, which can be viewed as the inverse process of polarization‐color imaging. We used an RGB color mixing method to achieve full‐color micro‐printing under a specific linearly polarized light. Each pixel in the original image was up‐sampled and decomposed into four subpixels: RGB and a non‐transmitting (black) subpixel. The RGB values of each subpixel were achieved by rotating the polarization orientation of the filters.

Since the average extinction ratio of the three filters exceeds 20 in their respective color bands, intensity‐polarization angle encoding can be applied based on Malus' law. Taking the red subpixel of the (*i*,  *j*) pixel as an example, the polarization orientation assigned can be expressed as:

(2) $$\theta_{R\left(i,j\right)}=\arccos{\left(I_{R}/I_{0}\right)}^{-\frac{1}{2}}$$

where *I_R_*/*I*_0_ = *R*_Value_/255. The normalized RGB values of the red pixel are selected, and we chose the fourth subpixel as black instead of green to fasten EBL writing and to simplify the RGB encoding process, as our goal here was to demonstrate the feasibility of color mixing. Based on Equation (2) and the image encoding workflow shown in Figure 5e, we produced two micro‐painting drawings: The Hammermen Crest (Figure 5f) that belongs to the Incorporation of Hammermen of Glasgow, a trades guild of which James Watt was a member. And the oil painting Girl with a Pearl Earring (Figure 5g) by Johannes Vermeer, the oil painting on canvas *Sunflowers* (F453) (Figure 5h) by Vincent van Gogh. The scale bars for all images are 100 µm, with each color subpixel sized at 2 µm.

Compared to the original images, the micro‐printed images appear darker overall, which is expected since we intentionally up‐sampled the image and introduced one black subpixel in each 4‐pixel RGB color unit, significantly reducing the overall brightness. Nevertheless, as a demonstration, the outlines and shading variations in the images are still clearly visible. Figure 5i shows a zoomed‐in view of the mixed‐color pixels, where the brightness variations of the RGB subpixels under 0° polarized light can be distinctly observed.

## 4 Conclusion

In conclusion, we proposed and fabricated an aluminum dimer aperture array plasmonic metasurface to exploit the extraordinary optical transmission effect, achieving efficient polarisation‐color filtering with CMOS‐compatible materials. To demonstrate the proposed filter's simultaneous polarisation‐color filtering capability, we demonstrated its application to structural color generation. The color palette generated by the structure covers ≈76% of the sRGB gamut, with blue (centered at 465 nm), green (centered at 540 nm), and red (centered at 638 nm) filters exhibiting FWHM of ≈75, 90, and 110 nm, and average extinction ratios exceeding 150, 100, and 20, respectively. Additionally, we demonstrated, based on the proposed structure, orthogonal linear polarisation‐encoded image encryption and micron‐scale full‐color reproduction of color‐rich images using RGB color mixing. The dimer structure we have demonstrated holds promise for applications in displays and imaging, particularly in multi‐dimensional light field detection when integrated with focal plane arrays.

## 5 Experimental Section

### Sample Fabrication

The fused quartz substrate was first cleaned sequentially in acetone and IPA solvent in a water bath for 5 mins of ultrasonic clean respectively, then 150 nm of aluminum was deposited using thermal evaporation. The 150 nm SiO2 was then deposited as a capping and adhesion‐improving layer using PECVD. After a further solvent clean, as described above, and a 10 min 180‐degree hot plate pre‐bake, 200 nm CSAR resist is spin‐coated on the cooled sample. A 60 nm charge dispersion layer of Electra 92 is then sputtered as a conductive layer to avoid charging in the top CSAR resist layer. Metasurface patterns are then written using a Raith EBPG 5200 EBL tool at a 100 keV accelerating voltage and 1 nA current. Samples were then developed in n‐Amyl acetate and IPA for 90 s, respectively. The dimer aperture patterns were then etched using an Oxford Instruments Plasmalab RIE 80 plus, (CH3F gas) for the SiO2 layers and a Plasmalab System100 ICP 180 (mixed Cl2/Ar gas) to dry‐etch the Al.

### Simulation

A 3D finite difference time domain (FDTD) method (ANSYS, Lumerical FDTD) was used to simulate the transmission spectrum of the monomer, dimer, and trimer arrays. A plane‐wave source, ranging from 400 to 1000 nm is set inside the substrate, and two perfectly matched layers (PML) are set at the top and bottom boundaries. Anti‐symmetric and symmetric boundary conditions are used for directions of the E‐field and M‐field of the plane wave, respectively, to save simulation time. The material database used for aluminum is the Palik database, while the refractive index for capping PECVD SiO2 is set as 1.46, according to the test result from the James Watt Nanofabrication Centre at the University of Glasgow. A monitor is set above the capping layer by 2 µm to eliminate any near‐field influence and capture spectral response. For angled incidence, the source is replaced with the broadband fixed angle source technique (BFAST), and the boundary conditions are correspondingly replaced with periodic boundaries. The color response derived from transmission spectra is calculated by decomposing it with CIE standard color‐matching functions. A flat‐emission light source is used for CIE color plot. The scripts partially use the file shared by the Matlab user community.^[^ ^50^ ^]^

### Spectral Characterization

A Foster‐Freeman micro‐spectrophotometer is used to optically characterize the spectral response of the fabricated sample. Condensers with fixed NA = 0.5 (f/# = 1) are used to test the filter performance under different objective lenses. A wire grid polariser (Thorlabs WP25M‐VIS) is placed in the light path to generate linear‐polarized incidence. A standard Halogen Light bulb is used as the illumination source, while the spectrum and color generated are normalized to the source spectrum.

Converting image to Ebeam readable files: Please refer to: https://github.com/FrankBiscuit/ConvertingImage2Ebeam

## Conflict of Interest

The authors declare no conflict of interest.

## Supporting information



Supporting Information

## Data Availability

The data that support the findings of this study are available from the corresponding author upon reasonable request.
