# Supplementary material for: Polarization‐Controlled Transmissive Plasmonic Color Filter Using a Dimer‐Aperture Array
Source: Adv Sci (Weinh). 2025 Mar 24;12(19):2501941. doi: 10.1002/advs.202501941 (PMC12097114; doi:10.1002/advs.202501941)
Supplement: Supplementary file 1 — Supporting Information [file ADVS-12-2501941-s001.docx]

**Supplementary Information for**

**Polarization-controlled Transmissive Plasmonic Color Filter Using a Dimer-Aperture Array**

Shuhao Wu^1^, Peter Connolly^2^, Vincenzo Pusino^1^, Gerald S. Buller^2^, David R.S. Cumming^1^*

1 *James Watt School of Engineering, Microsystem Group, University of Glasgow, G12 8QQ*

*2 School of Engineering and Physical Sciences, Heriot-Watt University, Edinburgh EH14 4AS, UK*

1. **Influence of geometrical parameter** $\boldsymbol{e}$ **and** $\boldsymbol{g}$

In geometric dimensions, the axial ratio $e=r1/r2$ primarily affects the anisotropic response of the metasurface to incident light. When the major axis, r1, of the ellipse remains unchanged (i.e., the ratio of $r1$ to the lattice constant $P$ is constant), as shown in Figure S.I. 1(a), the influence of $e$ within the range from 3.5 to 4.5 on the transmission spectrum at the maximum (TM illumination with respect to the X-Z plane) is small, with the peak transmission and line shape remaining largely unchanged. However, Figure S.I. 1(b) demonstrates that when we take the average extinction ratio (E.R.) in the green channel range (500–570nm), $e$ has a significant impact on the extinction ratio. A higher $e$ corresponds to a larger extinction ratio, which is primarily due to the increased difference in the grating-SPP interaction region under orthogonally polarized illumination.


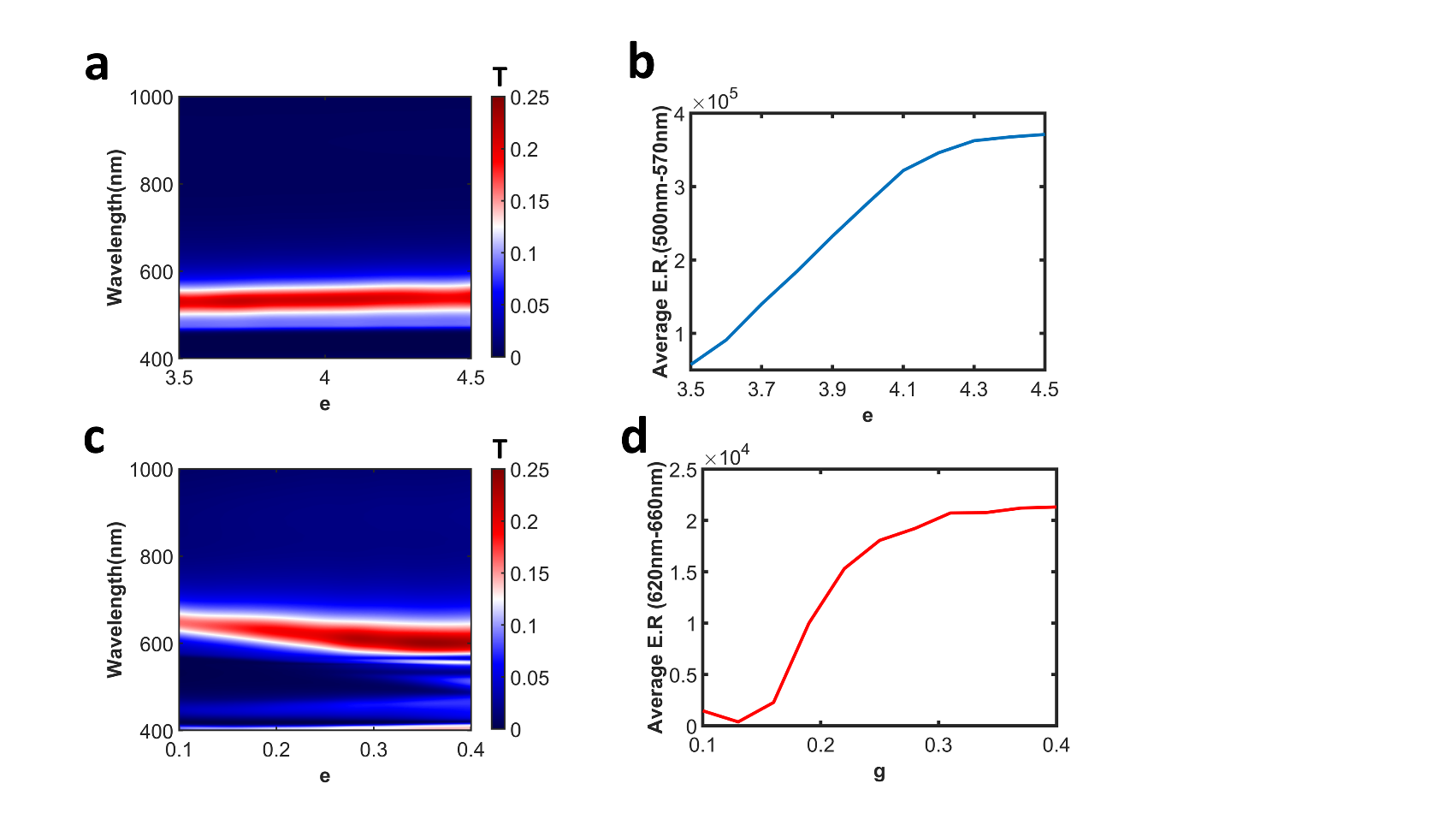


Figure SI.1 **a.** green channel filter transmission vs $e$. **b.** green channel filter average E.R. (within spectral range 500-570nm) vs $e$. **c.** red channel filter transmission vs $g$. **d.** red channel filter average E.R. (within spectral range 620-660nm) vs $g$.

As shown in Figures S.I. 1(c) and (d), the spacing between dimer-apertures has a notable effect. Taking the red channel as an example, smaller spacing results in a lower peak transmission and a significantly reduced extinction ratio. Increasing $g$ (see Section 2.1 of the main paper) leads to a higher extinction ratio and greater peak transmission but also results in a broader linewidth, enhanced higher-order modes, and a blue shift in the peak wavelength.

The blue shift of the peak wavelength associated with the structure implies that larger structures are required for the red channel, which in turn increases the minimum compatible pixel size for red filters. As a demonstration, we select $g$ = 0.2, which corresponds to a relatively narrow linewidth while ensuring at least three complete cycles in the x-y direction, respectively, within a 2-micron sized pixel.

1. **
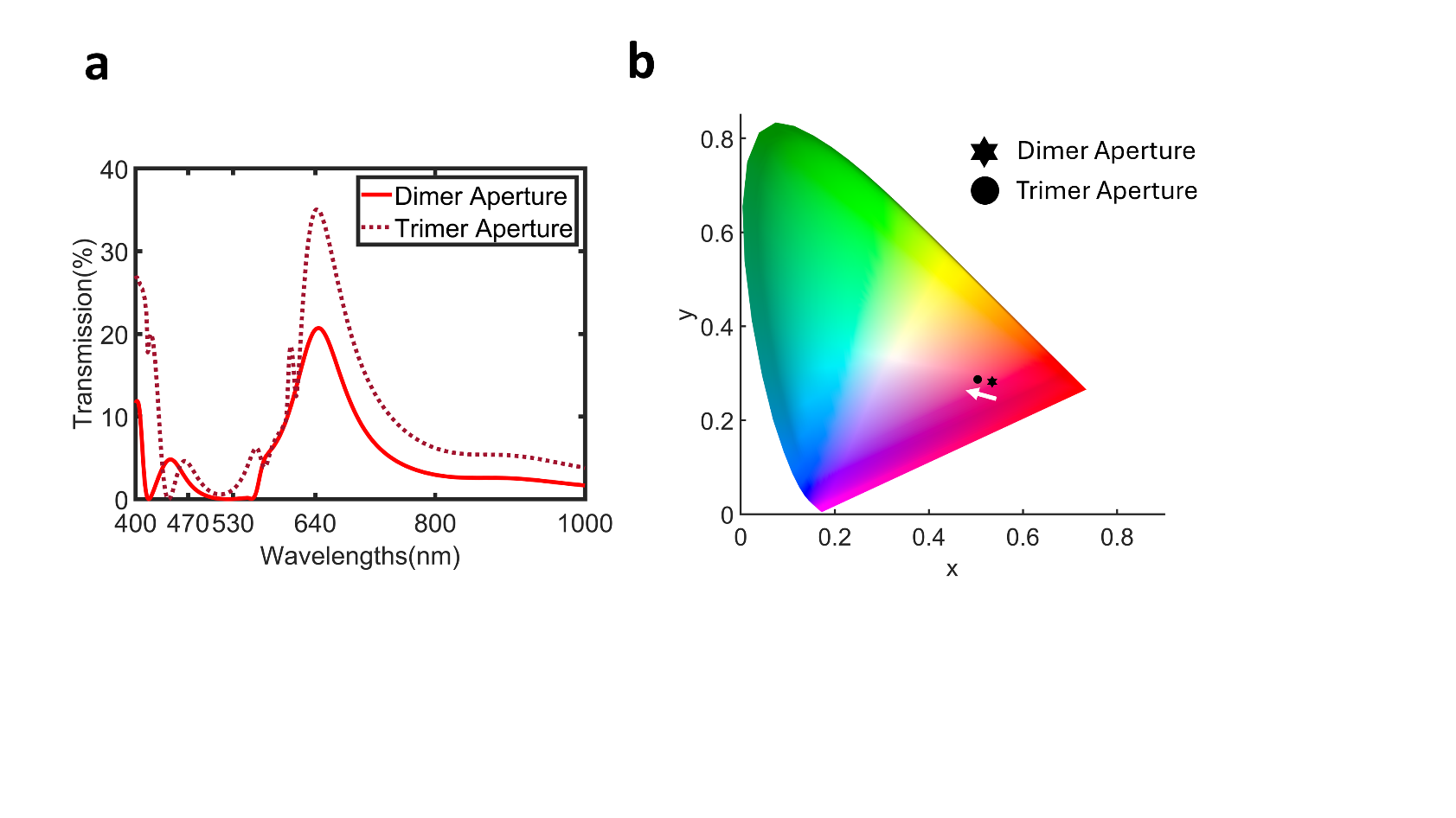
Comparison of trimer and dimer aperture configuration**

Figure SI.2 **a.** red channel filter transmission for dimer aperture configuration and trimer aperture configuration **b.** converted results of the transmission spectrum in **a** on CIE 1931 colour gamut.

For the blue and green channels, the trimer-aperture structure exhibits higher peak transmission and narrower linewidth. However, for the red channel, as is shown in Figure SI.2, the trimer structure demonstrates a noticeably poorer near-infrared (NIR) cutoff and more side bands. NIR cut-off is favoured for imaging applications, particularly for modern CMOS sensor chips that are responsive to near-infrared wavelengths. Therefore, we select the dimer structure as a demonstration to highlight the effects of multiple-aperture designs.

1. **
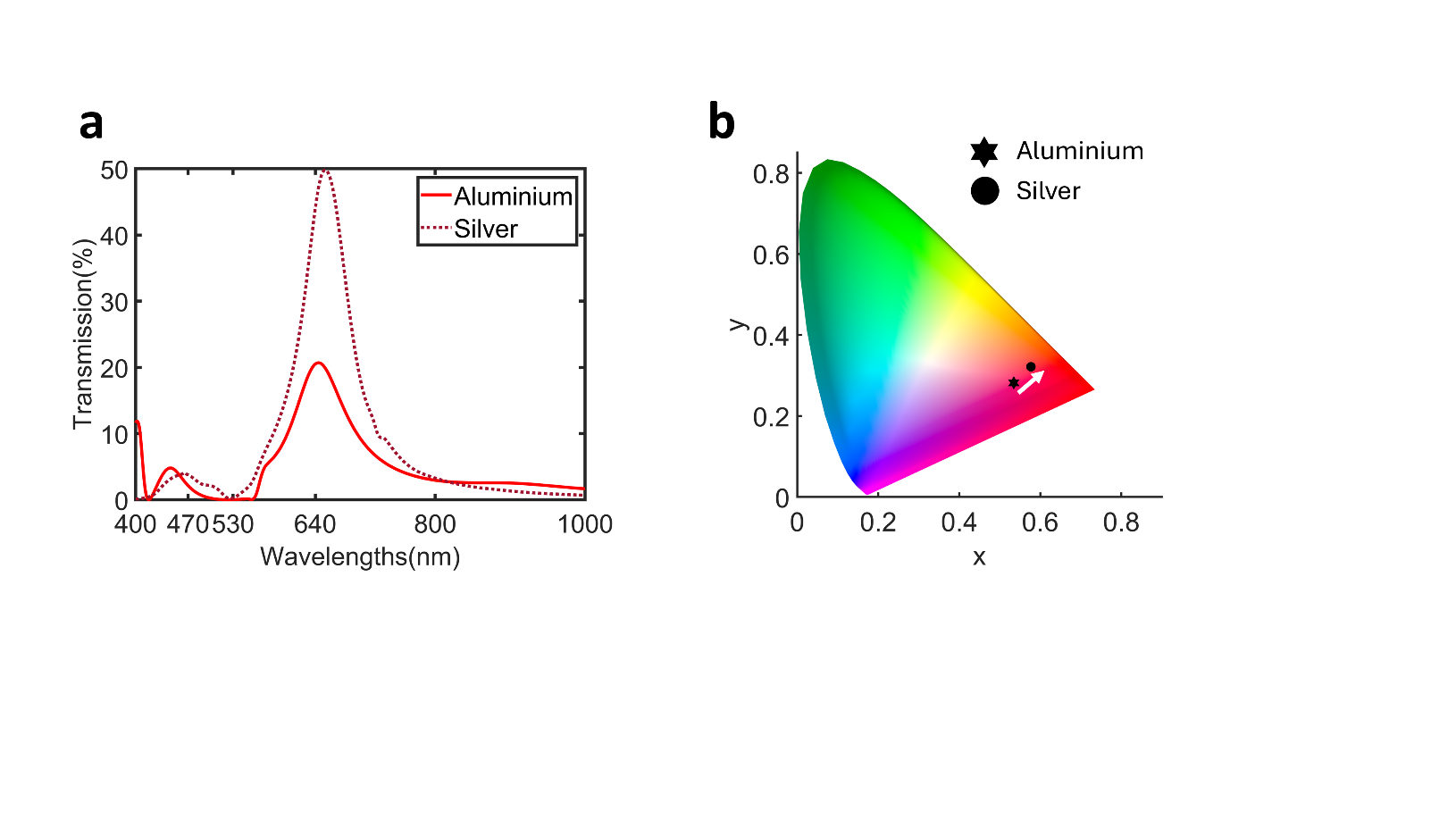
Efficiency enhancement with silver**

Figure SI.3 **a.** red channel filter transmission for comparison between aluminum aperture configuration and silver aperture configuration **b.** converted results of the transmission spectrum in **a** on CIE 1931 color gamut.

Due to lower loss features of silver compared to aluminum, a direct transmission coefficient enhancement method would be to replace the metal structure with silver. It can be clearly seen that the transmission can be enhanced to over 40% using same structure but using different dimensions compared to the structure in this work. However, silver suffers from the incompatibility to CMOS fabrication process, limiting its practical realization.

1. **SPP penetration depth estimation and substrate etching**

The penetration depth of SPP into a dielectric for a continuous substrate dielectric-metal interface without apertures can be estimated using the following equation:[1]:

$$\delta_{d}=\frac{1}{k_{0}}\left| \frac{\varepsilon_{m}^{'}+\varepsilon_{d}}{{\varepsilon_{d}}^{2}} \right|^{1/2}$$

where $\varepsilon_{m}^{'}$ represents the real part of the permittivity of the metal (aluminum in our case), and $\varepsilon_{d}$ refers to the permittivity of dielectric, which is silicon dioxide. For 530 nm wavelength, $\delta_{d}$ will be approximately 260 nm. Figure SI.4(a) shows the SPP depth into the substrate is approximately 150 nm under TM illumination. We found that within this length-scale, substrate etching can reduce the linewidth. From the depth-transmission spectrum, it can be observed that the linewidth decreases with increasing substrate etching depth, while the peak transmission varies within a 5% range. When the substrate etching depth exceeds 100 nm, the linewidth reduction plateaus. A substrate etching depth of 100 nm is therefore sufficient to achieve the desired effect.


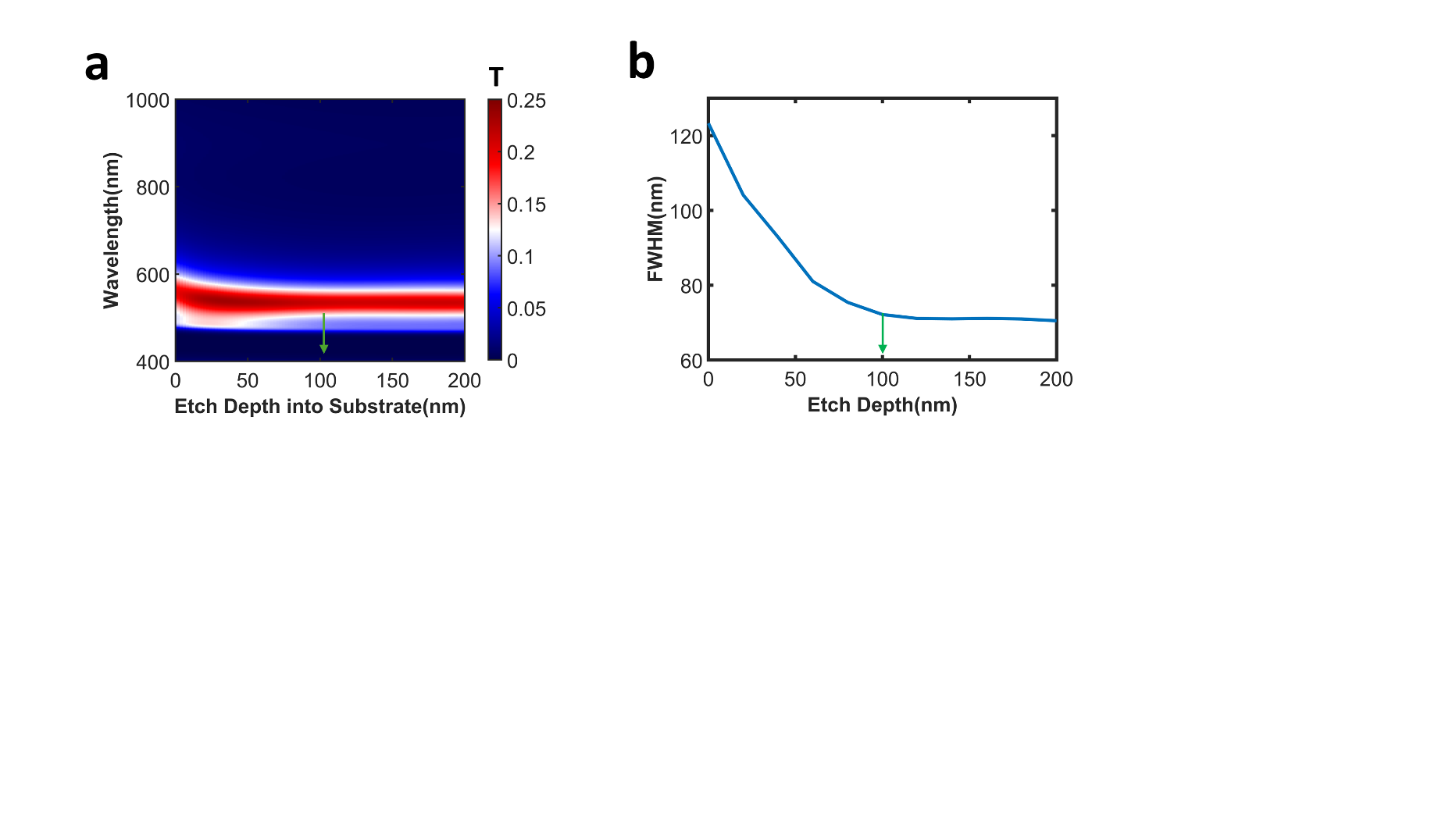


Figure SI.4 **a.** green filter transmission spectrum vs substrate etch depth **b.** FWHM of the filtering peak vs etch depth.

1. **Fabrication error and characterization**

The peak transmission and linewidth are larger than simulation results for filters. This is attributed to both excessive exposure doses and overly long development times, which result in enlarged apertures. When the aperture size increases, the non-frequency-selective direct transmission also increases, whereas SPP-related modes do not increase proportionally. Consequently, although the peak transmission increases, the frequency selectivity of the filter decreases. This leads to brighter images but a narrower color gamut. However, insufficient exposure or development can cause the apertures to remain unopened. Therefore, as a conceptual demonstration, we would rather use slightly higher doses and longer development times to ensure the basic function of the sample. Larger apertures correspond to a smaller extinction ratio. The extinction ratio is highly influenced by the axis ratio between long and short axis. For aperture shape, it would tend to be over-etched more in the direction along short axis (with narrower dimension) than along long axis (with wider dimension). Consequently, axis ratio between long and short axis are much smaller, as is shown in SI.5 (b). The design ratio should be 4 while the fabricated sample shows a ratio of approximately 3.1. Another major factor which contributes to the reduced extinction ratio is the spectrometer detection limit. We’ve set the illumination LED light at maximum, while the tool still cannot obtain stable readings for TE incidence. We therefore rounded all readings smaller than 0.1% to be 0.1%, which means
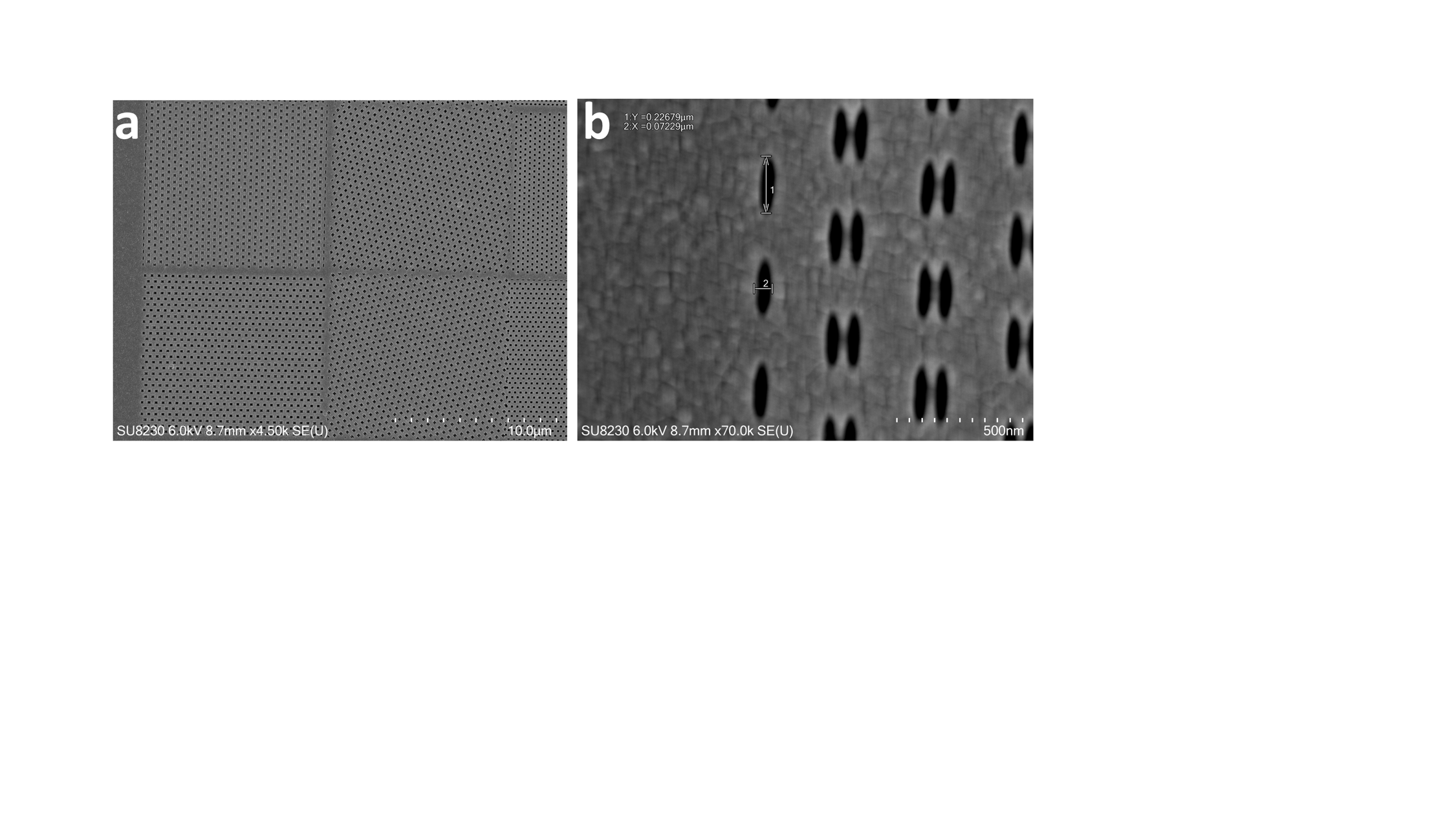
the E.R. ratio listed in the main text are only lower-limit estimates.

Figure SI.5 **a.** $\times50$k magnification SEM photo of the fabricated filters. **b.** $\times70$k magnification SEM photo of the fabricated red filters. The designed dimensions are 252nm and 63nm (ratio = 4) for long and short axis respectively, while the fabricated samples are 226 and 72nm (ratio=3.13) respectively.

1. Barnes, W.L., *Surface plasmon-polariton length scales: a route to sub-wavelength optics.* Journal of Optics a-Pure and Applied Optics, 2006. **8**(4): p. S87-S93.
